# Plasma-Derived Exosomal ALIX as a Novel Biomarker for Diagnosis and Classification of Pancreatic Cancer

**DOI:** 10.3389/fonc.2021.628346

**Published:** 2021-05-05

**Authors:** Jie Yang, Yixuan Zhang, Xin Gao, Yue Yuan, Jing Zhao, Siqi Zhou, Hui Wang, Lei Wang, Guifang Xu, Xihan Li, Pin Wang, Xiaoping Zou, Dongming Zhu, Ying Lv, Shu Zhang

**Affiliations:** ^1^ Department of Gastroenterology, Nanjing University Medical School Affiliated Nanjing Drum Tower Hospital, Nanjing, China; ^2^ Department of General Surgery and Pancreatic Disease Research Center, The First Affiliated Hospital of Soochow University, Suzhou, China

**Keywords:** exosomes, pancreatic cancer, proteomic profile, ALIX, diagnostic biomarker

## Abstract

**Background:**

Pancreatic cancer (PC) has a dismal prognosis due to its insidious early symptoms and poor early detection rate. Exosomes can be released by various cell types and tend to be a potential novel biomarker for PC detection. In this study, we explored the proteomic profiles of plasma exosomes collected from patients with PC at different stages and other pancreatic diseases.

**Methods:**

Plasma samples were collected from six groups of patients, including PC at stage I/II, PC at stage III/IV, well-differentiated pancreatic neuroendocrine tumor (P-NET), pancreatic cystic lesions (PCLs), chronic pancreatitis (CP), and healthy controls (HCs). Plasma-derived exosomes were isolated by ultracentrifugation and identified routinely. Isobaric tags for relative and absolute quantification (iTRAQ) based proteomic analysis along with bioinformatic analysis were performed to elucidate the biological functions of proteins. The expression of exosomal ALIX was further confirmed by enzyme-linked immunosorbent assay in a larger cohort of patients. Furthermore, receiver operating characteristic curve analysis was applied to evaluate the potential of ALIX as a novel diagnostic biomarker.

**Results:**

The proteomic profile revealed a total of 623 proteins expressed among the six groups, and 16 proteins with differential degrees of abundance were found in PC *vs.* other pancreatic diseases (including P-NET, PCLs, and CP). Based on the results of proteomic and bioinformatic analyses, exosomal ALIX was subsequently selected as a novel biomarker for PC detection and validated in another clinical cohort. We noticed that ALIX expression was elevated in PC patients compared with patients with other pancreatic diseases or HC, and it was also closely associated with TNM stage and distant metastasis. Interestingly, the combination of exosomal ALIX and serum CA199 has greater values in differentiating both early *vs.* late PC (AUC value 0.872) and PC *vs.* other pancreatic diseases (AUC value 0.910) than either ALIX or CA199 alone.

**Conclusion:**

In summary, our study demonstrated that based on proteomic profiling, proteins isolated from the plasma-derived exosomes may function as ideal non-invasive biomarkers for the clinical diagnosis of PC. Importantly, exosomal ALIX combined with CA199 has great potentials in detection of PC, especially in distinguishing PC patients at early stages from advanced stages.

## Introduction

As a kind of highly heterogenous malignancy, pancreatic cancer (PC) still faces great challenges in the early diagnosis and treatment options. The most recent data estimated by the American Cancer Society show that the incidence rates and death rates continue to increase for the cancer of pancreas (1). Due to the insidious early symptoms and rapid progression, more than 80% of PC patients are diagnosed at an advanced stage when the disease has been disseminated (1, 2). Nowadays, PC is commonly diagnosed by the combination of classical serum biomarkers (*e.g.* carbohydrate antigen 19-9, CA199), imaging examinations, and endoscopic biopsy. However, all these techniques are either invasive or unspecific. Yachida et al. (3) suggested that PC would take several years to develop from initial mutations to metastatic cancer, highlighting the significance of finding effective methods for early stage detection. Hence, it is very critical for us to develop new sensitive and non-invasive tools for PC diagnosis, and thus increase the early detection rate and overall survival rate for PC patients.

Recently liquid biopsy has been identified as a safer, faster, and non-invasive test compared with tissue biopsy (4, 5). Among various types of liquid biopsies, exosomes are one type of extracellular vesicles of 30–150 nm in diameter, originate from multi-vesicular endosomes, and are initially thought to be cellular debris (6, 7). Now exosomes are recognized and identified to be encompassed by a lipid bilayer membrane, containing specific proteins, nucleic acids, and lipids. They are secreted by diverse cell types and can be detected in most body fluids, so they can reflect the cell type of origin, mediate intercellular communication, and even shape the microenvironment of tumors (6–8). On account of this, exosome biomolecules have great potentials as non-invasive biomarkers for the diagnosis of PC at early stages, or therapeutic targets for the treatment of PC (9–11).

In the present study, we explored the proteomic profiles of exosomes in plasma samples collected from patients with PC (stage I/II and stage III/IV) and other pancreatic diseases, including chronic pancreatitis (CP), pancreatic cystic lesions (PCLs), and well-differentiated pancreatic neuroendocrine tumors (P-NETs). We aim to elucidate some specific exosomal proteins useful in the detection of PC.

## Materials and Methods

### Patients and Plasma Samples

This study included a total of 134 samples collected from patients with pancreatic diseases or healthy controls (HCs) who were admitted to the Nanjing Drum Tower Hospital Affiliated to Nanjing University Medical School and the First Affiliated Hospital of Soochow University between July 2018 and December 2019. Patients considered eligible for this study were those with suspected pancreatic masses and with operation indications. Endoscopic ultrasound-guided fine-needle aspiration (EUS-FNA) and/or surgical resections were performed for initial diagnosis. Plasma samples were all collected prior to EUS-FNA operations or surgical resections. All of them had not received any antitumor therapy before. Classification of PC was determined according to the 8^th^ edition of the American Joint Committee on Cancer (AJCC) Classification. In the discovery stage, plasma samples were selected from 30 subjects with PC (stage I/II and stage III/IV), well-differentiated P-NETs, PCLs, CP and HC (five cases in each group) from Nanjing Drum Tower Hospital. We pooled the samples from the same group into one representative sample with the purpose of increasing the efficiency of analysis and reducing the individual variations. In the validation cohort, in order to determine the diagnostic performance of candidate protein biomarkers, we enrolled another 104 subjects (18 patients with PC at stage I/II, 44 with PC at stage III/IV, 11 with PCLs, eight with well-differentiated P-NET, 13 with CP, and 10 with HC, respectively) from two hospitals. Plasma samples were prepared from EDTA-treated peripheral blood, centrifuged at 2,000×g and 10,000×g for 10 min (4°C) to remove dead cells and cell debris and stored at −80°C until subsequent analysis. The study was approved by the Ethics Committee of the Nanjing Drum Tower Hospital and the First Affiliated Hospital of Soochow University, and informed consent was obtained from all patients before the examinations.

### Exosomes Isolation

Exosomes were isolated by ultracentrifugation in accordance with the guidelines proposed by the International Society for Extracellular Vesicles (ISEV) (12). To isolate exosomes, the plasma was thawed on ice and centrifuged at 10,000×g for 30 min and then filtered through a 0.22-um filter (MilliPore, USA). The supernatant was then ultracentrifuged at 110,000×g for 70 min. The pallet was washed in phosphate-buffered saline (PBS) and centrifuged again at 110,000×g for 70 min. The PBS was removed completely, and subsequently 60 μl ice-cold PBS was used to resuspend the exosomal pellet. All centrifugation steps were performed at 4°C.

### Exosome Identification

General characterizations of exosomes were performed to demonstrate the purity of our exosome preparation. For images of exosomes at high resolution, about 5 μl of prepared exosomal suspension (the purified concentrated exosomes were mixed with an equal volume of 2% paraformaldehyde) was applied to the formvar-carbon coated grids, and the membranes were placed in a dry environment for 20 min. Then the grids were transferred to a 50 μl drop of 1% glutaraldehyde for 5 min and washed eight times with distilled waters. Samples were contrasted and embedded on ice with uranyl-oxalate for 5 min and methyl cellulose-uranyl acetate for 10 min, respectively. The excess fluid was blotted gently, and the grids were air dried for 10 min. The images were captured under the transmission electron microscopy (TEM, HT7800, Hitachi, Japan). Besides, the size distribution of exosomes was analyzed by high resolution flow cytometry using a Flow NanoAnalyzer Instrument (N30E, NanoFCM, China). The exosomal protein markers were validated by western blot analysis. We used the BCA protein assay kit (Thermo Fisher Scientific, USA) to quantify total protein concentration of exosomes according to the manufacturer’s protocol. The protein samples were denatured at 95°C for 10 min. Approximately 30 μg of protein was separated by 10% SDS-PAGE, subjected to the electrophoresis, and transferred to PVDF membranes. The membranes were then blocked with 5% skim milk for 2 h, incubated with anti-CD63 (Abcam, UK), anti-Tsg101 (Santa Cruz, TX), and anti-*β*-actin (Abcam, UK) overnight. Then protein bands were washed with tris-buffered saline with tween 20 (TBST) buffer three times and probed with secondary antibodies for 120 min.

### Exosomal Protein Lysis

Frozen exosomes were suspended on ice and 8 M urea was added. Then the samples were sonicated in ice for 2 min and incubated in ice for 30 min. We homogenized samples for 10 s every 5 min. The samples were clarified by centrifugation at 12,000×g at 4°C for 20 min. Protein was quantified with BCA protein assay kit (Thermo Fisher Scientific, USA). Approximately 30 μg of total protein was separated by 10% SDS-PAGE gel. Then separation gel was stained by CBB according to Candiano’s protocol. The stained gel was scanned by Image Scanner (GE Healthcare, USA) at the resolution of 300 dpi.

### Protein Digestion

A 200 ug portion of protein from each sample was reduced and alkylated in 50 mM tris-(2-carboxyethyl) phosphine (TCEP) at 56°C for 1 h and 200 mM methyl methanethiosulfonate (MMTS) at room temperature for 1 h. The chilled acetone was then added in the protein mixtures for precipitation overnight. The solutions were centrifuged at 30,000×g for 30 min at 4°C and dissolved in 0.5 M tetraethyl-ammonium bromide (TEAB) and sonicated in ice. Subsequently, trypsin was added to the samples at a 1:20 (w/w) ratio, and the solutions were incubated for digestion at 37°C for 16 h. Finally, the collections of digested peptides were centrifuged at 12,000 rpm for 20 min.

### iTRAQ-Labeling Proteomic Analysis

For iTRAQ-labeling, the lyophilized samples were resuspended in 100 μl TEAB (200 mM) and then labeled by 8-plex iTRAQ reagent (SCIEX, USA) according to the instructions in the kit (PC at stage III/IV with 113 and 114 tags, PC at stage I/II with 115 tag, P-NET with 116 tag, HC with 117 tag, PCLs with 118 tag, CP with 119 tag). The labeled peptides were finally dried for further analysis.

### Peptide Fractionation

The high pH reversed-phase liquid chromatography with a C-18 column was used to fractionate the labeled peptides. Briefly, mobile phases B (98% acetonitrile in 5 mM Ammonium formate) was used for gradient elution. The solvent gradient was set as follows: 0–25% B, 5–35 min; 25–45% B, 35–45 min. The peptides were separated at a fluent flow rate of 300 nl/min and finally lyophilized for mass spectrometry.

### Liquid Chromatography Coupled to Tandem Mass Spectrometry

LC-MS/MS analysis was performed on a Nanoeasy system (Thermo Fisher Scientific, USA) with a 25 cm-long column (75 μm × 25 cm, 2 μm, 100 Å, C18 packing material, Thermo Fisher Scientific, USA) connected to a Q Exactive hybrid quadrupole-Orbitrap mass spectrometer (Thermo Fisher Scientific, USA). The fractions were dissolved in loading buffer (2% acetonitrile with 0.1% FA) before analysis. Mobile phase B consisted of 98% acetonitrile and 0.1% formic acid. The elution gradient was set from 5 to 40%. Full MS scans were acquired in the mass range of 300–1200 m/z with a mass resolution of 70,000, and the AGC target value was set at 1e6. The ten most intense peaks in MS were fragmented with higher-energy collisional dissociation (HCD) with collision energy of 35. MS/MS spectra were obtained with a resolution of 17,500 with an AGC target of 1e5 and a max injection time of 22 ms. The Fusion dynamic exclusion was set for 60 s and run under positive mode.

### Database Search

The Proteome Discovery software (v.2.3) was used for protein identification and quantitation. All the raw data were searched against the Swiss-Prot human database (20,238 entries), with MS tolerance set at 20 ppm and MS/MS tolerance set at 0.1 Da. A global false discovery rate (FDR) was set to 0.01, and protein groups considered for quantification required at least two peptides.

### Bioinformatic Analysis

We obtained the information of identified proteins from the UniProt protein sequence database. Both Gene Ontology (GO) analysis and Kyoto Encyclopedia of Genes and Genomes (KEGG) pathway analysis were conducted for the function and pathway analysis of proteins by DAVID (https://david.ncifcrf.gov/) online tool. The STRING database was introduced for protein–protein interaction (PPI) network analysis (http://string-db.org).

### ELISA Procedures

The absolute expression levels of exosomal ALIX were tested using a commercial enzyme-linked immunosorbent assay (ELISA) kit (SenBeiJia Biotechnology Co., Ltd., China) based on the recommended manufacturer’s protocol. Firstly, the solubilized exosome samples were diluted five times with sample diluent, then 50 μl of the sample or standards was added to the appropriate wells in order. After the whole reaction, the optical density of each well was determined immediately using a microplate reader set to 450 nm. The concentration of exosomal ALIX was calculated in comparison with a protein standard curve.

### Statistical Analysis

All data were analyzed using the GraphPad Prism 6.0 statistical software (La Jolla, CA, USA) and SPSS 20.0 software (Chicago, IL, USA). The Mann–Whitney U test or Student’s t test was conducted to investigate the protein expression difference in all subjects. The expression levels of the selected biomarker candidate obtained by ELISA assays were compared using one-way ANOVA. The diagnostic value was evaluated by receiver operating characteristic (ROC) curve analysis. Differences were considered significant when *P <*0.05.

## Results

### Isolation and Characterization of Plasma-Derived Exosomes

**Figure 1:**
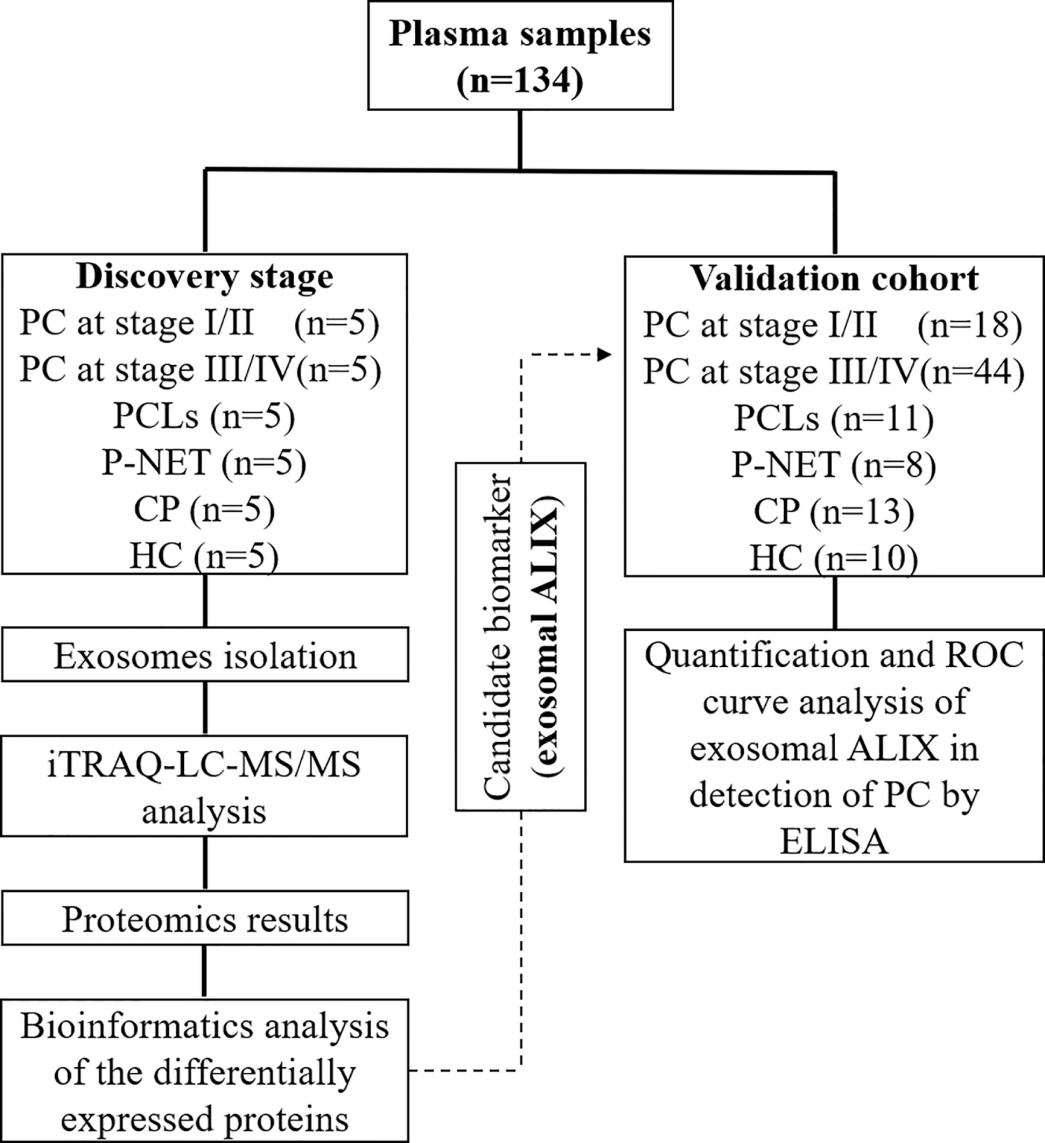
Schematic workflow of the study.

**Figure 2:**
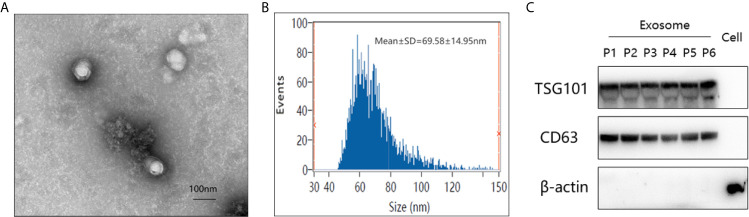
Verification of plasma-derived exosomes from pancreatic cancer patients. **(A)** Transmission electron micrograph of isolated exosomes. Scale bar = 100 nm. **(B)** Size distribution measurements of isolated exosomes by flow cytometry. **(C)** Western blot analysis of exosome-enriched proteins (Tsg101 and CD63) and a control marker (*β*-actin) in isolated exosomal proteins from six different groups of patients and the total protein from PANC-1 cells. (P1–6 represented patients from PC at stage I/II, PC at stage III/IV, well-differentiated P-NET, PCLs, CP, and HC, respectively).

The study workflow was shown in **Figure 1**. Firstly, the plasma-derived exosomes were isolated separately by ultracentrifugation. As shown in **Figure 2**, the structure of exosomes revealed as a cup-shaped, membrane-enclosed vesicles, with a 30–150 nm size range characterized by TEM and flow cytometry (**Figures 2A, B** **)**. The expression of exosomal biomarkers (CD63, Tsg101) was determined by western blotting using samples from six different groups of patients, which was shown in **Figure 2C**. And no significant difference in CD63 or Tsg101 intensity was observed among all groups of patients. Besides, exosomal biomarkers CD9, CD63, CD81, and Tsg101 were also all observed in the list of proteins identified through proteomic profiling (**Table S1**). In summary, these results absolutely confirmed the purity of plasma-derived exosomes used in this study.

### Proteomic Profiles of Plasma Exosomes

**Figure 3:**
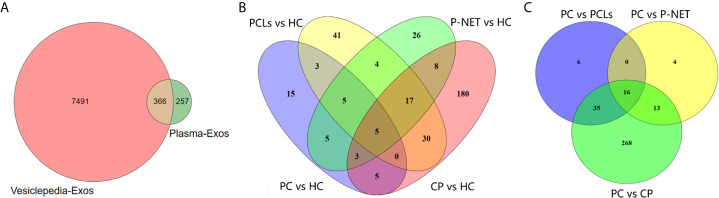
Differential proteomic analysis of plasma-derived exosomes from patients with PC, other pancreatic diseases and healthy individuals. **(A)** The Venn diagram depicted the overlap of proteins identified in present study with those published in the Vesiclepedia database. **(B)** The overlap between differentially expressed proteins in exosomes from PC, P-NET, PCLs, and CP groups *versus* HC group. **(C)** The overlap of identified proteins differentially expressed between PC and other pancreatic diseases.

In the discovery stage, exosomal proteins were derived from all six groups and detected for iTRAQ-LC-MS/MS proteomic profiling analysis. A total of 623 proteins were identified (**Table S1**), and 366 of them overlapped with the exosome database that was compiled in Vesiclepedia (**Figure 3A**). Moreover, based on the criteria for differentially expressed proteins (fold change >1.20 or fold change <0.83 with a relative quantification *P* value <0.05), 52 up-, 43 down- and six up-, 14 down-regulated proteins in exosomes were discovered from PC stage I/II and stage III/IV patients compared with healthy groups. Besides, 73, 105, and 248 proteins were found to be differentially expressed in exosomes from P-NET, PCLs, and CP groups *versus* HC group (**Figure 3B**). In addition, 22 proteins showed significant differential expression between stage I/II and stage III/IV PC patients. Apart from this, exosomes from P-NET, PCLs, and CP groups had differential expression of 33, 57, and 332 proteins in comparison with PC, respectively (**Figure 3C**). When we compared the identified proteins between PC and other pancreatic diseases groups, we noticed an overlap of 16 proteins, which were presented in **Table 1**.

**Table 1:** An overlap of 16 differentially expressed proteins between the exosomes of PC and other pancreatic diseases.

Uniprot accession	Gene symbol	Protein name	Protein abundances ratio *(PC*/*other pancreatic diseases)*
P01024	C3	Complement C3	1.179
A0A075B7D0	IGHV1OR15-1	Immunoglobulin heavy variable 1/OR15-1	0.6249
Q8WUM4	PDCD6IP	Programmed cell death 6-interacting protein	1.8389
Q9NZH0	GPRC5B	G-protein coupled receptor family C group 5 member B	1.7719
G5EA09	SDCBP	Syndecan binding protein (Syntenin), isoform CRA_a	1.5119
P53990	IST1	IST1 homolog	1.5269
P02538	KRT6A	Keratin, type II cytoskeletal 6A	0.2459
P04259	KRT6B	Keratin, type II cytoskeletal 6B	0.2339
P68871	HBB	Hemoglobin subunit beta	0.655
P69905	HBA1	Hemoglobin subunit alpha	0.621
P02533	KRT14	Keratin, type I cytoskeletal 14	0.315
P08779	KRT16	Keratin, type I cytoskeletal 16	0.441
Q04695	KRT17	Keratin, type I cytoskeletal 17	0.270
P01762	IGHV3-11	Immunoglobulin heavy variable 3-11	0.971
P35443	THBS4	Thrombospondin-4	1.045
A0A140TA62	N/A	IF rod domain-containing protein	0.699

PC, pancreatic cancer.

### Identification of Differentially Expressed Proteins in Plasma-Derived Exosomes

**Figure 4:**
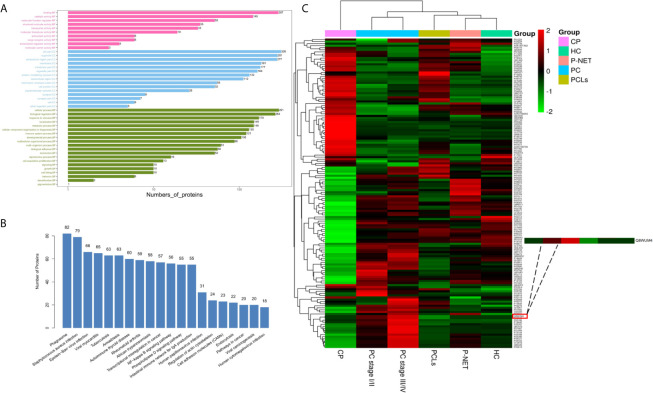
Biological function and quantitative evaluation of plasma-derived exosomal proteins. **(A)** Gene ontology (GO) classification of whole identified exosomal proteins. The enriched terms in the biological process, cellular component, and molecular functions were listed. **(B)** Kyoto Encyclopedia of Genes and Genomes (KEGG) pathway analysis of whole identified exosomal proteins. The top 20 enriched pathways in exosomes were listed. **(C)** Hierarchical clustering heat maps of 150 important proteins, which were identified by t-test. The expression of each protein was illustrated in red and green to indicate high and low expression, respectively.

Bioinformatic analysis was performed to reveal the functional characterization of the differentially expressed proteins. GO annotations indicated that protein binding, cell part, and cellular process were the most enriched terms in the molecular function (MF), cellular component (CC), and biological process (BP) (**Figure 4A**). KEGG pathway analysis showed that many reported signaling pathways associated with tumorigenesis and metastasis in PC, including transcriptional mis-regulation in cancer, endocytosis, and NF-kappa B signaling pathway were all included in the top 20 enriched pathways (**Figure 4B**). Moreover, an intensity heatmap was also utilized to depict the proteins that were significantly differentially expressed among the six groups and identified in the Uniprot and Vesiclepedia database (150 proteins in total) (**Figure 4C**).

**Figure 5:**
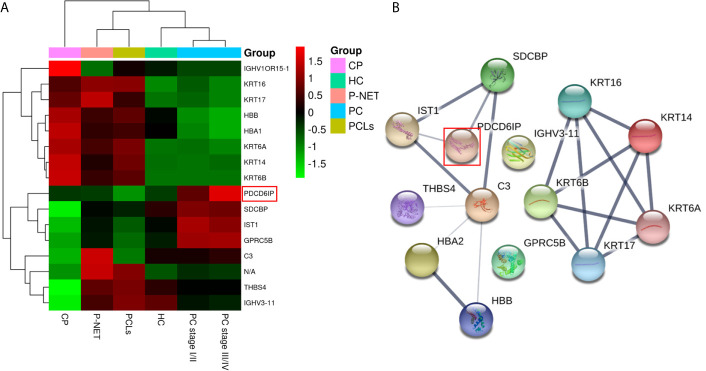
Hierarchical clustering analysis and protein–protein interaction network (PPI) analysis of the 16 overlapped exosomal proteins. **(A)** Heatmap of the 16 proteins differentially expressed between the exosomes of PC and other pancreatic diseases. **(B)** The STRING PPI network of 16 differentially expressed proteins. It yielded a highly clustered network containing 14 nodes with 16 edges (clustering coefficient: 0.738, enrichment p-value < 0.001).

Among all differentially expressed proteins, there were a total of 16 proteins that overlapped between PC and other pancreatic diseases groups. Four proteins were significantly higher in PC compared with other pancreatic diseases, including PDCD6IP (also known as ALIX), GPRC5B, SDCBP, and IST1 (**Figure 5A**). To reveal the relationships of the 16 proteins, we reconstructed the interaction networks using the STRING protein–protein interaction (PPI) database. The STRING PPI analysis yielded a highly clustered network containing 14 nodes with 16 edges (clustering coefficient: 0.738, enrichment p-value < 0.001). As shown in **Figure 5B**, among the four upregulated proteins in PC group, three were interacted with each other (ALIX, SDCBP and IST1), and the fold change of ALIX expression in PC *vs.* other pancreatic diseases was higher compared with both SDCBP and IST1 (**Table 1**). Based on the above results, we subsequently selected exosomal ALIX as the candidate biomarker for further analysis.

### Validation of Exosomal ALIX as a Novel Biomarker for Pancreatic Cancer

**Figure 6:**
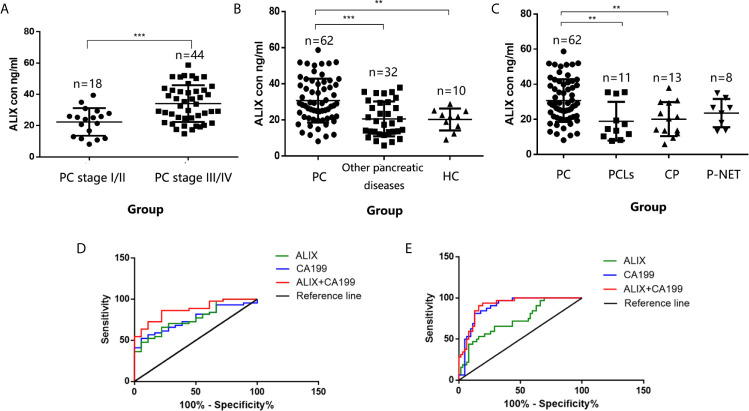
Validation of exosomal ALIX as a novel biomarker for pancreatic cancer diagnosis and classification. **(A)** Scatter dot plots of protein concentrations of exosomal ALIX in patients with PC at different stages analyzed by ELISA. **(B)** Scatter dot plots of protein concentrations of exosomal ALIX in patients with PC, other pancreatic diseases, and healthy controls. **(C)** Scatter dot plots of protein concentrations of exosomal ALIX in patients with PC compared with all other types of pancreatic diseases. **(D)** ROC curve analysis of exosomal ALIX and serum CA199 for discrimination between PC patients at stage I/II and stage III/IV. **(E)** ROC curve analysis of exosomal ALIX and serum CA199 in differentiating PC *vs.* other pancreatic diseases. **P < 0.01; ***P < 0.001.

Late diagnosis and lack of effective treatments are the main reasons for poor prognosis of PC. Hence, we focused on investigating the diagnostic utility of exosomal ALIX as a novel biomarker for PC with plasma samples from patients with PC, other pancreatic diseases, and healthy controls. The expression level of exosomal ALIX was evaluated by ELISA, and ROC curve analysis was performed to assess the diagnostic value of ALIX. A total of 104 individuals were enrolled in this clinical cohort, including 62 patients with PC (stage I/II, n = 18; stage III/IV, n = 44), 11 patients with PCLs, eight patients with well-differentiated P-NET, 13 patients with CP, and 10 healthy subjects. At the same time, the general clinicopathological features were observed and summarized. We noticed that the exosomal ALIX expression was significantly higher in advanced PC than early PC (**Figure 6A**). Moreover, ALIX expression was significantly elevated in all PC patients compared with patients with other pancreatic diseases in total or HC (**Figure 6B**). Interestingly, we also found that the expression of exosomal ALIX in PC patients was higher compared with other pancreatic diseases, including PCLs (*P* = 0.0037), CP (*P* = 0.0043) and P-NET (*P* = 0.1132) separately, though there was no statistically significant difference between PC and P-NET (**Figure 6C**). Next, we analyzed the correlations between clinical characteristics and exosomal ALIX expression level in PC patients by univariate analysis, and the results showed that ALIX expression was significantly associated with TNM stage (I/II *vs.* III/IV) and distant metastasis (*P* < 0.05) (**Table 2**). Then, we explored the value of exosomal ALIX alone or in combination with serum CA199 in differential diagnosis of pancreatic diseases (**Table 3**). For one thing, ALIX presented an acceptable diagnostic efficacy with an AUC value of 0.730 between PC and other pancreatic diseases, which was a liter lower than CA199 (0.891). For another, when differentiating PC patients at stage I/II from stage III/IV, ALIX had a slightly higher AUC value (0.768) than CA199 (0.756). We further combined exosomal ALIX with CA199 to estimate their diagnostic performance in differentiating PC *vs.* other pancreatic diseases, as well as early *vs.* late PC. Consequently, the combination of these two markers for the diagnosis of PC *versus* other pancreatic diseases yielded a promising AUC of 0.910, with a sensitivity of 90.6% and specificity of 83.9% at the cut-off point (**Table 3**, **Figure 6E**). They also improved the diagnostic efficacy between patients at stage I/II and stage III/IV with the AUC value of 0.872, which was significantly higher than CA199 alone (**Table 3**, **Figure 6D**). Therefore, we suggested that exosomal ALIX had the capability to detect PC, and the combination of ALIX and CA199 can further improve the diagnostic value, especially in distinguishing early and late PC.

**Table 2:** Relationship between exosomal ALIX expression and clinicopathological parameters in PC groups.

	Total (n = 62)	ALIX expression		*P* value
		Low	High	
Sex				0.309
Male	30	13	17	
Female	32	18	14	
Age (years)				0.611
<65	30	16	14	
≥65	32	15	17	
Smoking status				0.520
Smoker	12	7	5	
Non-smoker	50	24	26	
Drinking status				0.354
Drinker	5	4	1	
Non-drinker	57	27	30	
Obstructive jaundice				0.562
Positive	16	7	9	
Negative	46	24	22	
Diabetes				0.576
Positive	18	8	10	
Negative	44	23	21	
TNM				0.011^*^
I/II	18	14	4	
III/IV	44	17	27	
Metastasis				0.007^*^
Positive	21	5	16	
Negative	41	26	15	

PC, pancreatic cancer; TNM, T, extent of the primary tumor; N, lymph node involvement; M, metastatic disease. *P < 0.05.

**Table 3:** Diagnostic performance of exosomal ALIX and serum CA199 in distinguishing PC *vs.* patients with other pancreatic diseases and PC patients at different stages.

	Biomarker	AUC value	95%CI	*P* value	Sensitivity	Specificity
**PC *vs.*** **other pancreatic diseases**	ALIX	0.730	0.624–0.836	0.0003	53.1%	83.9%
	CA199	0.891	0.825–0.956	<0.0001	81.3%	87.1%
	ALIX+CA199	0.910	0.853–0.968	<0.0001	90.6%	83.9%
**PC stage I/II** ***vs.*** **PC stage III/IV**	ALIX	0.768	0.647–0.889	0.001	65.9%	77.8%
	CA199	0.756	0.638–0.875	0.002	52.3%	94.4%
	ALIX+CA199	0.872	0.785–0.960	<0.0001	86.4%	77.8%

PC, pancreatic cancer.

## Discussion

Due to the lack of an effective diagnostic method, the majority of PC patients are still diagnosed at an advanced stage and succumb to death within 12 months of diagnosis (1, 2, 13). Thus, it is urgent for us to seek more valuable biomarkers for early detection of PC. The present study investigated the differentially expressed exosomal proteins extracted from the plasma of patients with PC, other pancreatic diseases, and HC. The approach of iTRAQ-based proteomic analysis was used in the study, and a total of 623 proteins were identified. Bioinformatic analysis also indicated that the differentially expressed proteins were mainly located in the extracellular region and membrane parts and presented with a variety of extracellular functions in the processes of tumor progression and metastasis. Among the 16 differentially expressed proteins overlapped between PC and other pancreatic diseases, we selected exosomal ALIX as the candidate biomarker for further investigation. We found that ALIX protein expression, especially combined with serum CA199, had great potentials in differentiating PC patients at stage I/II from stage III/IV, as well as distinguishing PC from other pancreatic diseases.

Nowadays, PC still remains one of the deadliest cancer types worldwide, and the prominent problem is that no reliable markers are recommended for routine screening because of insufficient sensitivity and specificity. Therefore, exploring a precise and non-invasive biomarker is very critical for the early detection of PC. In recent years, extracellular vesicles (EVs), a heterogeneous group of cell-derived membranous structures, have gotten more and more attention (6–8). Exosomes belong to the group of EVs and can mediate as a functional mediator (12, 14). Up to now, numerous studies have reported that exosomes are qualified as tumor specific biomarkers (9–11, 15–18). For one thing, as a bridge of communication, exosomes can assist the information exchange among cells in the form of lipids, nucleic acid species, and proteins, so the cargo of exosomes can reflect the real origin of them. For another, the lipid bilayer membrane can prevent exosomes from being degraded and keep information stable. More importantly, exosomes are released by various types of cells and can be simply isolated from different kinds of body fluids, such as blood, urine, saliva, and breast milk. Additionally, Jody et al. (19) compared the identified plasma-derived EVs against those found in unfractionated peripheral plasma and found that 41% proteins had not been previously identified in the latter group, indicating that some useful proteomic signatures may be missed by conventional profiling of total plasma. Based on the special characteristics of exosomes mentioned above, we presume that the plasma-derived exosomes can be an important noninvasive biomarker for early detection of PC.

As we all know, a great number of studies have been performed over the years on the diagnostic value of exosomes as a non-invasive biomarker for PC. Melo et al. (9) identified that GPC1^+^ circulating exosomes (crExos) could serve as a potential diagnostic and screening tool to detect early stages of PC to facilitate possible curative surgical therapy. Though several follow-up studies queried the effect of GPC1^+^crExos in the diagnosis of PC (16, 20, 21), the diagnostic value of exosomes still cannot be denied. In 2018, utilizing *in vitro* cellular assays, Jin et al. (22) found that exosomal ZIP4 can significantly promote PC growth, thus confirming the efficacy of ZIP4 as a novel diagnostic biomarker for PC. However, no clinical data have been published to verify the diagnostic value of exosomal ZIP4. Yu et al. (11) provided the first genome-wide analysis of extracellular vesicle long RNAs (exLRs) in plasma from PC patients, demonstrating the feasibility of identifying cancer biomarkers based on exLR profiling. These studies can fully confirm the value of exosomes as an ideal non-invasive biomarker for tumor detection. Unfortunately, no exosomal protein markers have been widely accepted for early detection of PC. Increasing researches have utilized proteomic analysis to discover new biomarkers recently, and iTRAQ is one of the most widely used techniques in the field of quantitative proteomics (23–26), which possesses the advantages of high throughout, high stability, and maximum protein coverage. Koichiro et al. (27) conducted a proteomic analysis using iTRAQ to explore proteins associated with lymph node metastasis in patients with colorectal cancer and identified 60 differentially expressed proteins. An et al. (28) performed the proteomic analysis in the serum-derived exosomal proteins and detected approximately 800 exosomal proteins in each incurably PC patients. In this work, we systemically studied the plasma-derived exosomal proteins and their biological functions in patients with pancreatic diseases and healthy individuals. Exosomes were purified by ultracentrifugation, and iTRAQ labeling couple LC-MS technique was used to evaluate the qualities of exosomal proteins. Out of the total 623 identified proteins, 366 proteins overlapped in the Vesiclepedia protein list. Different from previous studies, broader and larger groups of patients were enrolled in present study, including PC, well-differentiated P-NET, PCLs, CP, and healthy controls. These results are more authentic, providing a basic foundation for our further studies. 16 proteins with differential degrees of abundance were found in exosomes of PC *versus* other pancreatic diseases. Some of them were upregulated in patients with other pancreatic diseases, such as HBB, HBA1, and KRT16 proteins, while some were upregulated only in PC groups (*i.e.* ALIX, GPRC5B, SDCBP, and IST1), highlighting their potential diagnostic value. Moreover, 22 proteins were significantly differentially expressed between PC patients with stage I/II and stage III/IV. GO annotations indicated that protein binding in MF, cell part in CC and cellular process in BP were the most enriched terms. KEGG pathway analysis showed that many reported signaling pathways associated with tumorigenesis and metastasis in PC were all included in the top 20 enriched pathways.

Generally, exosomes are secreted upon fusion of endosomal multivesicular bodies (MVBs) with the plasma membrane, and the endosomal sorting complex required for transport (ESCRT) associated protein ALIX (also referred to as PDCD6IP, protein accession: Q8WUM4) plays an important role in the mechanisms involved in their biogenesis (29). Previous studies have identified that ALIX is a cytoplasmic protein, involved in endocytosis, membrane repair and the pathway of selected sorting by ESCRT-complexes (30, 31). Moreover, several researches have described that ALIX participates in programmed cell death, and its overexpression may block apoptosis (30, 32). As a general marker of exosomes, ALIX has scarcely been reported as a tumor marker, and the relationship between exosomes and protein degree of abundance has not been validated. Diederick et al. (33) compared exosomes from non-cancerous prostate cell lines to exosomes from prostate cancer (PCa) cell lines and finally identified ALIX as being enriched in PCa exosomes. What’s more, Monypenny et al. (34) confirmed that the ESCRT-related protein ALIX could regulate tumor-mediated immunosuppression by controlling EGFR activity and PD-L1 presentation. In our study, according to the proteomic results, we noticed that exosomal ALIX was significantly highly expressed in PC patients, especially in those at the advanced stage. Bioinformatic analysis also revealed that ALIX was related to protein binding, apoptotic process, and the pathway of endocytosis, indicating ALIX as a potentially diagnostic marker for PC. Hence, we performed ELISA experiments and demonstrated that ALIX expression was obviously higher in PC than in patients with other pancreatic diseases or healthy controls and was closely associated with TNM stage and distant metastasis. Besides, ALIX expression was significantly enhanced in late PC compared with early PC. Interestingly, combination of exosomal ALIX and serum CA199 has greater values in distinguishing both early *vs.* late PC and PC *vs.* patients with other pancreatic diseases than either ALIX or CA199 alone.

Certain limitations should be considered in the current study. Firstly, the sample size for candidate biomarker validation was not big enough, so we need to conduct a larger and multicenter trial to elucidate the diagnostic value of exosomal ALIX in the next phase. Secondly, we only selected an upregulated protein as the novel biomarker, ignoring the diagnostic value of proteins downregulated in PC groups. What’s more, although previous studies have confirmed that exosomes can be purified well enough by ultracentrifugation, we still cannot rule out the possibility that some information of proteins was lost in the process of exosome isolation.

## Conclusion

In conclusion, we provided a systematic approach for screening exosome-derived biomarkers for PC detection. Our study revealed that based on the proteomic profiling, plasma-derived exosomal proteins may function as ideal non-invasive biomarkers for early detection of PC. Importantly, exosomal ALIX combined with CA199 has great potentials in detection of PC, especially in distinguishing PC patients at early stages from advanced stages. And the function and molecular mechanisms of exosomal ALIX in PC progression need to be further investigated.

## Data Availability Statement

The raw data supporting the conclusions of this article will be made available by the authors, without undue reservation.

## Ethics Statement

The studies involving human participants were reviewed and approved by the Ethics Committee of the Nanjing Drum Tower Hospital and the First Affiliated Hospital of Soochow University. The participants provided their written informed consent to participate in this study.

## Author Contributions

JY and XG wrote the manuscript. JY, YZ, and YY performed the experiments. JY, SiZ, LW, and PW performed the statistics analysis. YY, JZ, and HW collected the samples and implemented the data acquisition. GX, XL, and ShZ revised the manuscript. YL, ShZ, and DZ contributed to the conception, design, and supervision of the study. All authors contributed to the article and approved the submitted version.

## Funding

This work was supported by the National Natural Science Foundation of China (81802396), Natural Science Foundation of Jiangsu Province (SBK2019022491 & BK20180117), General Project of Nanjing Medical Science and Technology Development Project (YKK17077), Nanjing Science and Technology Development Plan Project (201715023), Nanjing Medical Science and Technology Development Key Project (ZKX18022), and the Nanjing Science and Technology Project (201911038).

## Conflict of Interest

The authors declare that the research was conducted in the absence of any commercial or financial relationships that could be construed as a potential conflict of interest.

## Supplementary Material

Supplementary Table 1 A list of total proteins identified in the plasma-derived exosomes.
